# Identification of amitriptyline HCl, flavin adenine dinucleotide, azacitidine and calcitriol as repurposing drugs for influenza A H5N1 virus-induced lung injury

**DOI:** 10.1371/journal.ppat.1008341

**Published:** 2020-03-16

**Authors:** Fengming Huang, Cong Zhang, Qiang Liu, Yan Zhao, Yuqing Zhang, Yuhao Qin, Xiao Li, Chang Li, Congzhao Zhou, Ningyi Jin, Chengyu Jiang

**Affiliations:** 1 Hefei National Laboratory for Physical Sciences at the Microscale and School of Life Sciences, University of Science and Technology of China, Anhui, China; 2 State Key Laboratory of Medical Molecular Biology, Institute of Basic Medical Sciences Chinese Academy of Medical Sciences, Department of Biochemistry, School of Basic Medicine Peking Union Medical College, Beijing, China; 3 Genetic Engineering Laboratory, Institute of Military Veterinary Medicine, Academy of Military Medical Sciences, Changchun, China; University of Georgia, UNITED STATES

## Abstract

Infection with avian influenza A H5N1 virus results in acute lung injury (ALI) and has a high mortality rate (52.79%) because there are limited therapies available for treatment. Drug repositioning is an economical approach to drug discovery. We developed a method for drug repositioning based on high-throughput RNA sequencing and identified several drugs as potential treatments for avian influenza A H5N1 virus. Using high-throughput RNA sequencing, we identified a total of 1,233 genes differentially expressed in A549 cells upon H5N1 virus infection. Among these candidate genes, 79 drug targets (corresponding to 59 approved drugs) overlapped with the DrugBank target database. Twenty-two of the 41 commercially available small-molecule drugs reduced H5N1-mediated cell death in cultured A549 cells, and fifteen drugs that protected A549 cells when administered both pre- and post-infection were tested in an H5N1-infection mouse model. The results showed significant alleviation of acute lung injury by amitriptyline HCl (an antidepressant drug), flavin adenine dinucleotide (FAD; an ophthalmic agent for vitamin B2 deficiency), azacitidine (an anti-neoplastic drug) and calcitriol (an active form of vitamin D). All four agents significantly reduced the infiltrating cell count and decreased the lung injury score in H5N1 virus-infected mice based on lung histopathology, significantly improved mouse lung edema by reducing the wet-to-dry weight ratio of lung tissue and significantly improved the survival of H5N1 virus-infected mice. This study not only identifies novel potential therapies for influenza H5N1 virus-induced lung injury but also provides a highly effective and economical screening method for repurposing drugs that may be generalizable for the prevention and therapy of other diseases.

## Author summary

Highly pathogenic avian influenza (HPAI) A virus H5N1 causes acute lung injury (ALI) and acute respiratory distress syndrome (ARDS), with mortality as high as 52.79%. No vaccine for HPAI virus is available, and current treatments for influenza A H5N1 virus-induced ALI have limitations. Drug repurposing may be an effective approach for developing novel therapeutic strategies. In this study, we identified 4 drugs, the antidepressant amitriptyline HCl, the ophthalmic flavin adenine dinucleotide, the anti-neoplastic azacitidine and the vitamin D-deficiency treatment calcitriol, as being highly effective for the treatment of H5N1 virus-induced ALI using a transcriptomic-based high-throughput repurposing drug screening. These approved drugs might constitute novel potential remedies for treating influenza H5N1 virus infection, and this screening method may be generalizable for drug repositioning to identify new indications for other diseases.

## Introduction

Drug discovery obeys Eroom’s law: innovation slows as cost increases. Since 2000, the cost of developing a new drug has exceeded 1 billion USD, and this number continues to rise[1]. Drug repositioning, which identifies new indications for existing drugs, is an alternative approach that is both more efficient and economical because the safety profiles of the drug candidates are known. A well-known example is aspirin: it was originally marketed for its analgesic, antipyretic and anti-inflammatory effects but later was widely used to treat stroke and myocardial infarction (MI) based on its inhibition of platelet aggregation. According to Bernard Munos, a member of the National Center for Advancing Translational Sciences (NCATS) at the NIH, up to 75% of known drugs may be suitable for drug reposition[2].

H5N1 is a highly pathogenic avian influenza A virus known to cause acute lung injury (ALI) and acute respiratory distress syndrome (ARDS), with estimated mortality as high as 52.79%[3, 4]. Because current treatments for influenza virus infection and ARDS still present limitations, drug repurposing may be an effective method to identify novel therapeutic strategies to treat H5N1 virus-induced respiratory injury.

Previous studies from our laboratory have shown that H5N1-induced ALI in a mouse model can be alleviated by each of the traditional cardiovascular medicines losartan (an angiotensin II receptor blocker) and recombinant human angiotensin-converting enzyme 2 (hACE2) and the antimalaria drug chloroquine (an autophagy inhibitor)[5–7]. It was further shown that monoclonal antibodies against C-X-C motif chemokine 10 (CXCL-10 or IP-10) or interleukin 17A (IL-17A), used to treat immune system diseases, were able to ameliorate ALI induced by swine-origin influenza A H1N1 in mice[8, 9]. All of these drugs were identified based on an understanding of the molecular mechanism of ALI. Although a few studies have been performed based on a group of genes, their results were not confirmed by well-designed experiments[10–12]. Thus, a genomic approach to identify drugs for potential repurposing is worth exploring, and in this work, we propose a highly efficient, genome-wide method to identify drugs to be repurposed for the treatment of ALI induced by H5N1 influenza virus infection.

## Results

### Screening of repurposing drugs against H5N1 infection in A549 cell lines by RNA sequencing

**Fig 1:**
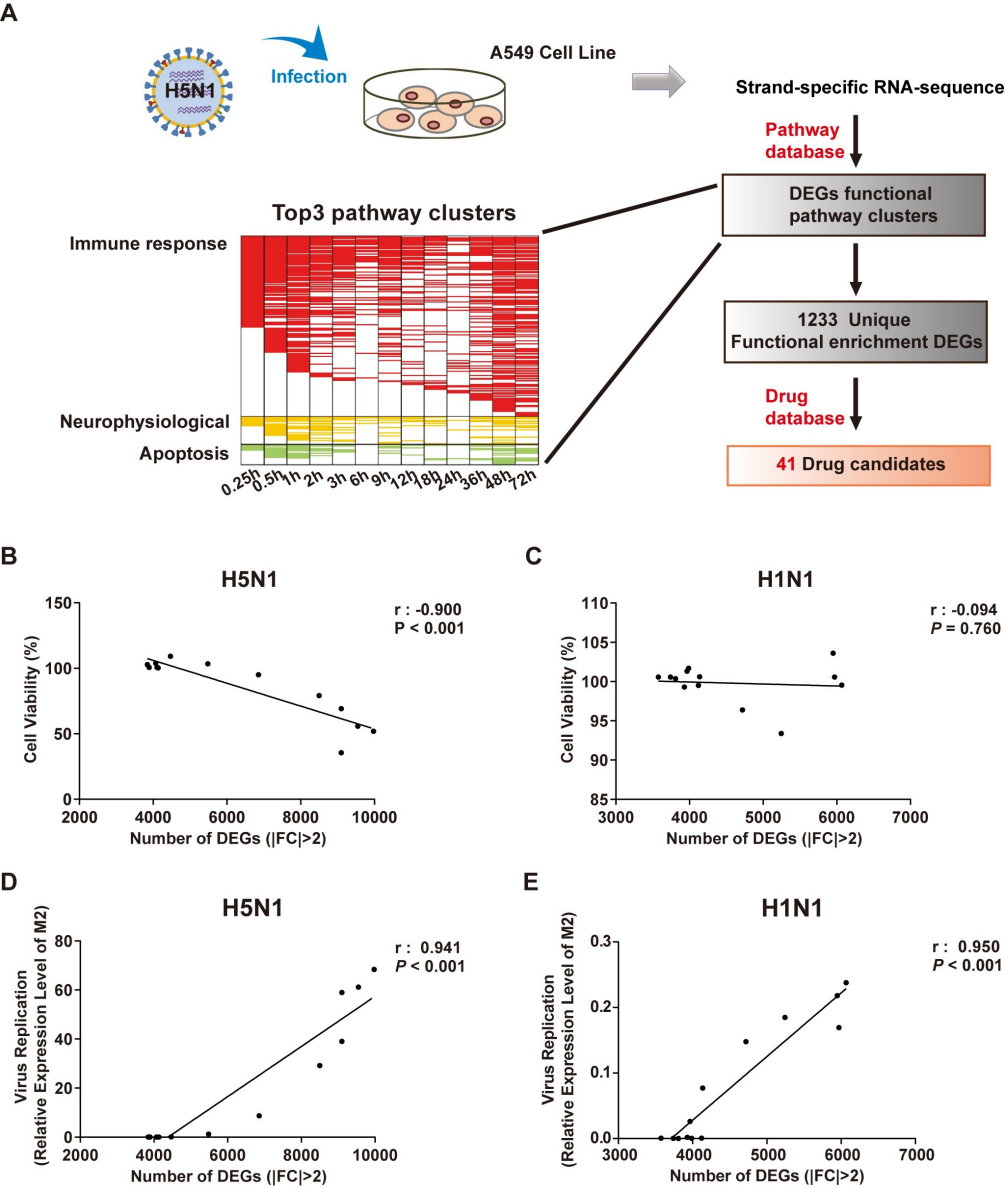
Screening of candidate drugs against H5N1 infection. **(A)** Schematic diagram of strand-specific RNA sequencing for drug candidate selection and functional enrichment pathways of differentially expressed genes at 0, 15 min, 30 min, 1 h, 2 h, 3 h, 6 h, 9 h, 12 h, 18 h, 24 h, 36 h, and 48 h after H5N1 infection in A549 cells. The heatmap shows pathways related to immune responses (red), neurophysiology (yellow), and apoptosis (green), with a two-tailed P value < 0.05 and multiple-testing Benjamini & Hochberg correction < 0.05. **(B-E)** Correlation between the numbers of DEGs in H1N1/H5N1-infected and control cells. (**B, C**) Cell viability based on the MTS assay. (**D, E**) Virus replication based on M2 expression by RT-PCR. Cell viability and viral replication were measured at 0 h, 15 min, 30 min, 1 h, 2 h, 3 h, 6 h, 9 h, 12 h, 18 h, 24 h, 36 h, and 48 h after H5N1 or H1N1 infection. The Pearson correlation coefficient (r) and P value are provided in the graph. DEG, differentially expressed genes; FC, fold change.

We infected A549 human lung carcinoma cells with lethal avian influenza A H5N1 virus or low-virulence seasonal influenza A H1N1 virus. Cells exposed to allantoic fluid (AF) were included as an additional control. Samples were collected at a series of time points for strand-specific total RNA-seq (Fig 1A). We identified differentially expressed genes (DEGs) between uninfected and infected cells (greater than 2-fold change) and calculated the linear correlation between the number of DEGs and cell viability and the level of virus replication. The number of DEGs altered by H5N1 infection, but not H1N1 infection, correlated significantly with cell viability (Fig 1B and 1C). The numbers of DEGs also correlated linearly with virus replication for both H5N1 and H1N1 (Fig 1D and 1E).

Pathway enrichment analysis using the Metacore database[13] indicated that most pathways in H5N1/H1N1-infected A549 cells are related to immune response, with neurophysiological and apoptotic processes being the second and third largest groups among the clustered pathways (Figs 1A and S1 and S1 Table). We filtered 1,233 unique DEGs included in all functional enrichment pathways that were altered by H5N1 infection (Figs 1A and S1). Using the DrugBank database[14] (with a total of 1645 drug target genes) to discover potential drug targets among the DEGs, we identified 79 drug target genes and 59 approved drugs that might be effective against H5N1 infection (Figs 1A and S1 and S2 Table). We obtained 41 commercially available drugs for biological confirmation.

### Measuring the efficacy of repurposing drugs in A549 cells (*in vitro*) and in mice (*in vivo*)

**Fig 2:**
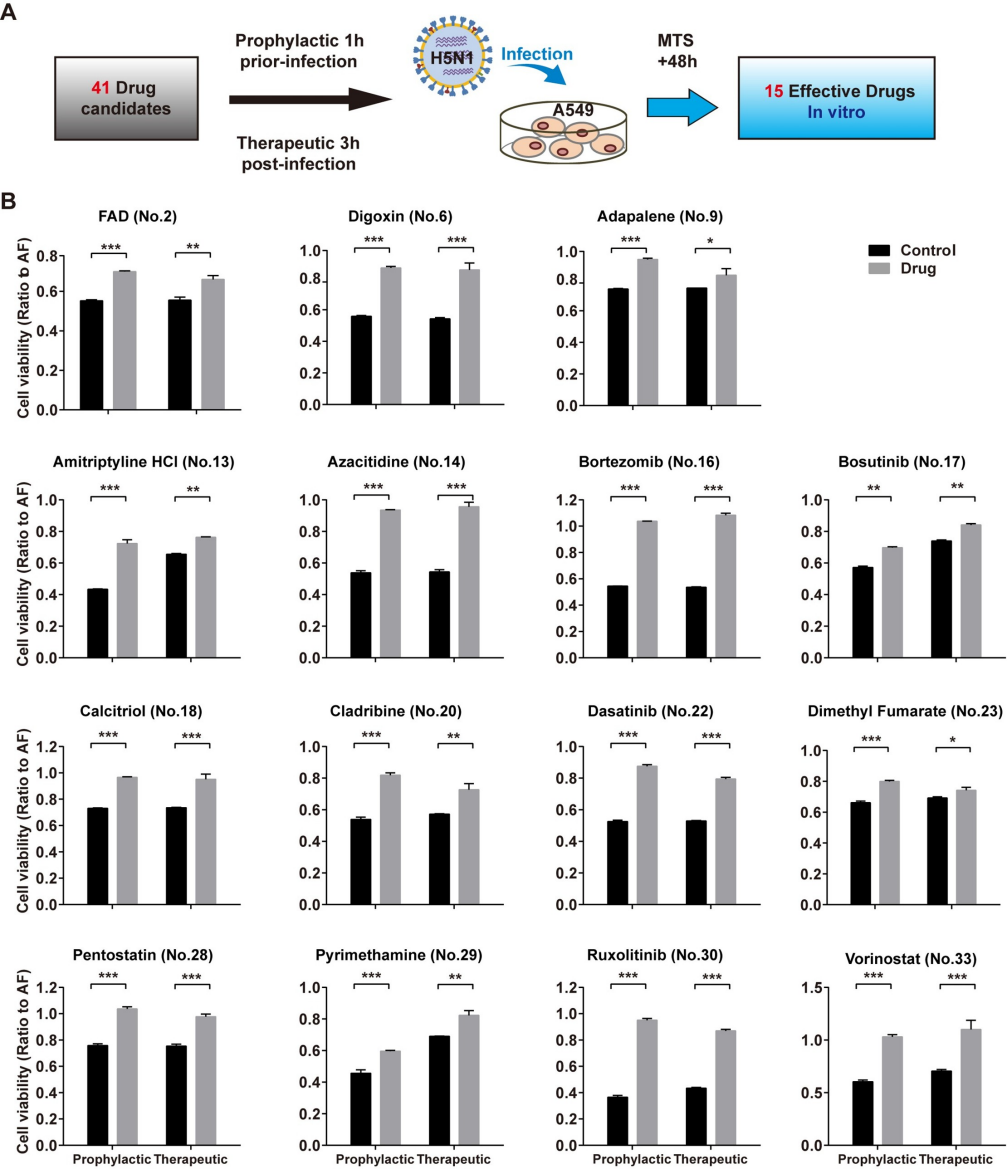
*In vitro* validation of candidate drugs against H5N1 in the A549 cell line. **(A)** Flowchart of screening for drugs against H5N1 infection in A549 cells. **(B)** Viabilities of A549 cells based on the MTS assay at 48 h after H5N1 virus infection. Cells were treated with drug or vehicle (control) either at 1 h before infection or at 3 h after infection. Data are presented as the mean ± SEM. All experiments were repeated at least twice. *P<0.05, **P<0.01, ***P<0.001 (two-tailed multiple comparison t-test with Holm-Sidak method, n = 3 biological replicates). Detailed information about *in vitro* drug treatment is shown in S2 Table.

We screened 41 drug candidates identified by high-throughput screening for potential prophylactic and therapeutic efficacy in H5N1-infected A549 cells (Fig 2A and S2 Table). The results confirmed that 22 drugs increased cell viability prophylactically or therapeutically, 15 of which were most effective both prophylactically and therapeutically (Figs 2B and S2).

**Fig 3:**
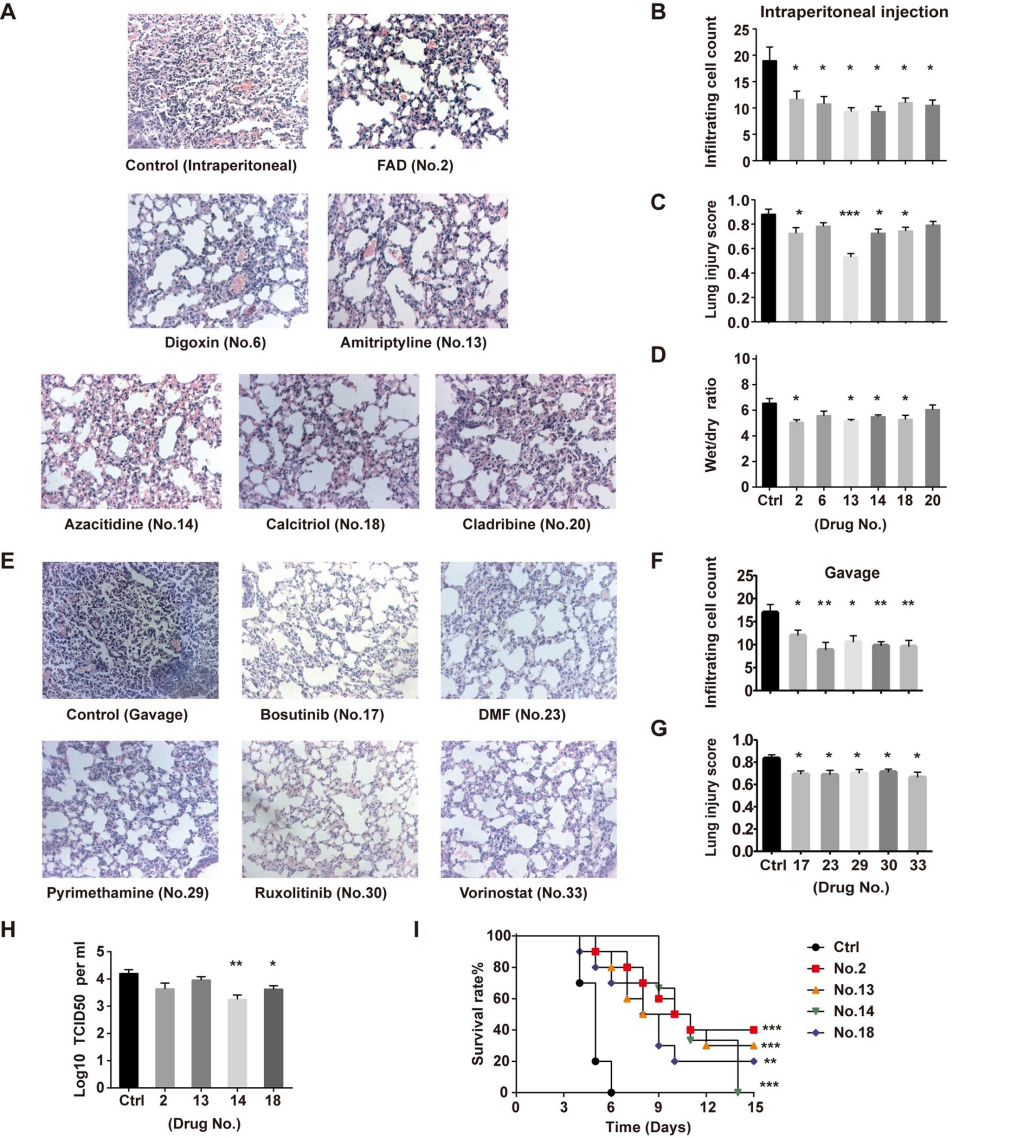
*In vivo* validation of candidate drugs against H5N1 in mice. Animals were infected with H5N1 (10^6^ TCID_50_) by intratracheal instillation and treated with drug intraperitoneally or gavage and then analyzed at 3 d after infection. (**A**) Images of lung pathology in mice following drug treatment by intraperitoneal injection. Magnification, 200×. For each treatment, 100 fields were analyzed (n = 4–6 mice per group). (**B**) Infiltrating cell numbers and (**C**) lung injury scores per microscopic field (mean ± SEM) are shown in the bar graphs. (**D**) Wet to dry weight ratios (mean ± SEM) of mouse lungs at 3 d after infection with drug treatment intraperitoneally. **(E)** Images of lung pathology in mice following drug treatment by gavage. Magnification, 200×. For each treatment, 100 fields were analyzed (n = 4–6 mice per group). (**F**) Infiltrating cell numbers and (**G**) lung injury scores per microscopic field (mean ± SEM) are shown in the bar graphs. **(H)** Viral titers of mouse lungs (mean ± SEM) are expressed as TCID_50_ per milliliter (n = 4–5 mice per group). All experiments were performed at least twice. *P<0.05, **P<0.01, ***P<0.001 (two-tailed one-way ANOVA). **(I)** Kaplan-Meier survival curves of H5N1-infected C57BL/6 mice treated with FAD (n = 10), amitriptyline HCl (n = 10), azacitidine (n = 6), and calcitriol (n = 10) or vehicle (n = 10) by intraperitoneal injection. **P<0.01, ***P<0.001 (log-rank test).

We then examined the efficacy of the 15 drugs in H5N1-infected mice (S3A Fig and S2 Table). The results showed that amitriptyline HCl, flavin adenine dinucleotide (FAD), azacitidine and calcitriol significantly decreased inflammatory cell infiltration, reduced lung injury scores, and ameliorated lung edema, as based on decreasing the wet-to-dry weight ratio of the lung tissue (Figs 3A–3G and S3B). We measured the viral load in mouse lung tissues and found that both azacitidine and calcitriol significantly inhibited H5N1 virus replication (Fig 3H). In addition, we evaluated the impact of the four candidate drugs on mouse survival after H5N1 virus infection. Notably, amitriptyline HCl, FAD and calcitriol significantly increased the survival rates of H5N1-infected mice, and azacitidine significantly prolonged survival time (Fig 3I). In the amitriptyline HCl, FAD and calcitriol administration groups, the body weight of H5N1-infected mice was recovered at the second week post infection (S3C Fig).

### Elucidating the molecular mechanisms of identified drugs

To further confirm the efficacy of amitriptyline HCl, FAD, azacitidine and calcitriol, we obtained RNA-seq data from H5N1-infected mouse lung tissue samples treated with drug vs. vehicle, identified DEGs and used the Metacore database to perform process and pathway enrichment for functional analysis (S4 Fig).

**Fig 4:**
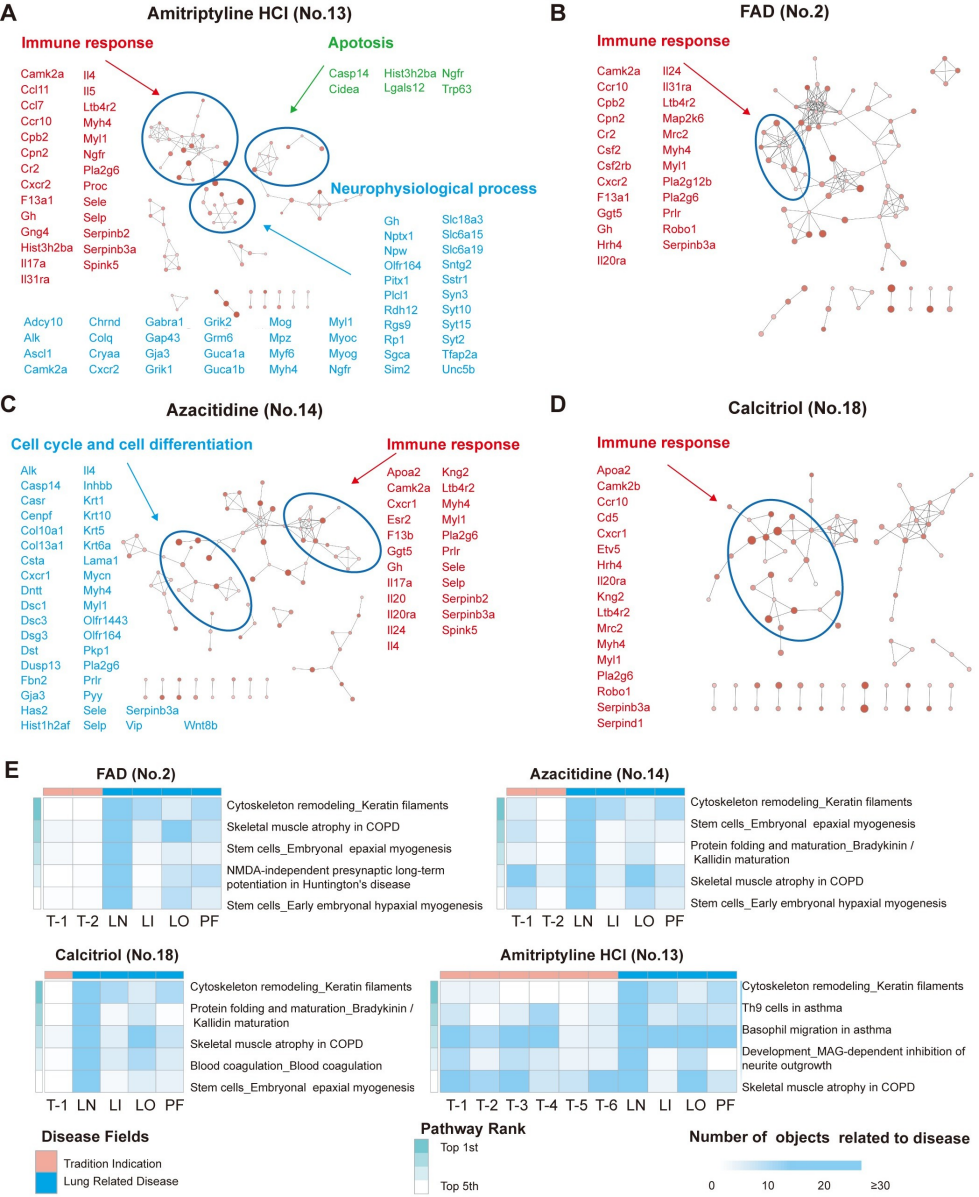
Functional processes and pathways influenced by amitriptyline HCl, FAD, azacitidine and calcitriol in H5N1-infected mice. Animals (n = 3–5) administered drugs were infected with H5N1 (10^6^ TCID_50_) by intratracheal instillation. Lung tissues were sampled for RNA-seq analysis at 2 d after H5N1 infection. Functional processes and pathways from DEGs influenced by drug administration were enriched by Metacore. **(A-D)** Process network enrichment of DEGs influenced by **(A)** amitriptyline HCl (no. 13), **(B)** FAD (no. 2), **(C)** azacitidine (no. 14) and **(D)** calcitriol (no. 18) treatment in H5N1-infected mice. Cytoscape with the Enrichment Map application was used for visualization. Nodes represent enrichment process networks; connections indicate shared objects between process networks. DEGs involved in the process networks are shown in the figure. The significance level and the object count enriched in the processes are reflected by the node color and node size, respectively. **(E)** Heatmaps of RNA sequencing data showing the numbers of objects related to traditional drug indications or a repurposed indication of lung-related disease in functional enrichment pathways of mouse lung tissue. Pathways with a two-tailed P value < 0.05 and multiple-testing Benjamini & Hochberg correction < 0.05 were considered significant. Abbreviations: LN, lung neoplasm; LI, lung disease (interstitial); LO, lung disease (obstructive); PF: pulmonary fibrosis; T, traditional indication-related disease. Detailed information about pathways and diseases related objects in the pathways is shown in S3–S6 Tables.

Amitriptyline HCl is an antagonist of the alpha-2A adrenergic receptor (ADRA2A), which belongs to the G protein-coupled receptor family, and is used clinically as a neural system drug to treat depression. A previous study has reported that ADRA2A blockade attenuates lung injury in rats [15], and our RNA-seq data showed significantly increased ADRA2A levels in H5N1-infected A549 cells. Analysis of the RNA-seq data from H5N1-infected mouse lung tissues indicated that genes significantly influenced by amitriptyline HCl treatment mainly clustered into immune responses, neurophysiological processes and apoptosis-related processes (Fig 4A). An investigation of the functions of these genes showed dozens in the top five pathways to be linked to both lung disease and to the traditional neural system indications of amitriptyline HCl (Fig 4E and S3 Table), suggesting that amitriptyline HCl ameliorates H5N1-induced ALI in mice.

FAD is an ophthalmic agent approved for the treatment of vitamin B2 deficiency. Previous studies have reported that riboflavin (vitamin B2) attenuates lipopolysaccharide (LPS)-induced lung injury in rats; inhibition of thioredoxin reductase 1 (TXNRD1), a target of FAD, attenuates lung injury and improves survival in murine models through the Nrf2 pathway [16–18]. In our experiment, H5N1 virus infection elevated the level of TXNRD1 in A549 cells, and FAD altered levels of immune response-related genes in the lungs of H5N1-infected mice (Fig 4B). Dozens of genes in the top five pathways are reported to be associated with lung disease as well as the traditional indication related to vitamin B deficiency therapy (Fig 4E and S4 Table). We determined that FAD is effective against avian influenza A H5N1 virus infection and ameliorates lung injury.

Azacitidine is an inhibitor of DNA (cytosine-5)-methyltransferase 1 (DNMT1) used to treat malignant tumors. In A549 cells, DNMT1 expression was significantly elevated by H5N1 virus infection. Previous studies showed that DNMT1 inhibition activates the Stat3 pathway and reduces LPS-induced ALI in mice[19, 20]. Calcitriol is an active form of vitamin D3 used to treat vitamin D deficiency. Activation of vitamin D receptor (VDR) signaling has been shown to attenuate LPS-induced ALI in mice[21]. By analyzing clusters of DEG functions in H5N1-infected mouse lung tissue, we found that azacitidine influenced the immune response as well as cancer mechanisms, including cell cycle and cell differentiation; calcitriol also influenced the immune response (Fig 4C and 4D). Dozens of genes among the top five pathways with changes in response to azacitidine or calcitriol are reported to be linked to lung disease in addition to their traditional indication: an anti-neoplastic indication for azacytidine and vitamin D deficiency-related osteoporosis for calcitriol (Fig 4E and S5 and S6 Tables). Previous studies reported that azacitidine and calcitriol alleviate LPS-induced ALI in mice[20, 22], and the results from the current study extend such findings to H5N1-induced ALI in mice.

We also analyzed pathway enrichment of the DEGs altered by the other seven drugs that decreased inflammatory cell infiltration in the lungs of H5N1-infected mice (Figs 3B–3F and S4B). In addition to their association with traditional disease indications, dozens of genes in the top five most significant pathways of each drug group are highly related to lung disease, including lung neoplasm, interstitial lung disease, obstructive lung disease or pulmonary fibrosis (S4B Fig and S7–S13 Tables).

Taken together, the results of the current study suggest that the neural system drug amitriptyline HCl, ophthalmic drug FAD, anti-neoplastic drug azacitidine and vitamin D deficiency treatment drug calcitriol may be novel treatments for severe avian influenza virus-induced lung injury. Azacitidine and calcitriol were previously reported to attenuate LPS-induced ALI in mice; these results indicate amitriptyline HCl and FAD as novel potential therapies to ameliorate lung injury.

## Discussion

In this study, we identified drugs effective for the treatment of lung injury caused by avian influenza infection using a transcriptomic-based high-throughput repurposing drug screen. We not only found four drugs effective *in vivo* that were able to counteract avian influenza H5N1 virus-induced lung injury but also identified seven drugs able to decrease inflammatory cell infiltration in the mouse lung following H5N1 infection and increase the viability of H5N1-infected cells both prophylactically and therapeutically (Figs 2B and 3). Our results suggest that lung injury can potentially be treated with four anti-cancer agents (bosutinib, cladribine, ruxolitinib, vorinostat), one immunoregulatory agent (dimethyl fumarate; DMF), one cardiovascular medicine (digoxin) or one antimalarial medicine (pyrimethamine).

The MAPK signaling pathway is involved in both the inflammatory response and lung injury[23, 24]. The expression level of mitogen-activated protein kinase kinase kinase 2 (MAP2K2), the target of bosutinib, a drug approved for the treatment of chronic myelogenous leukemia (CML), was significantly altered in cultured cells infected with H5N1 in the current study. Alleviation of lung injury by bosutinib might involve the MAPK signaling pathway via MAP2K2 inhibition. The Jak/Stat signaling pathway is an important pathway related to lung injury[25, 26], and in this study, we found Jak1/2 to be among DEGs in A549 cells infected with H5N1. We therefore speculate that the mechanism by which ruxolitinib, an inhibitor of Jak1/2 approved for myclofibrosis treatment, ameliorates H5N1-induced lung injury occurs via the Jak/Stat signaling pathway. A previous study showed that histone deacetylase (HDAC) inhibitors attenuate ALI in mice[27], and HDAC2/6 has been implicated in the therapeutic effects of vorinostat for the treatment of cutaneous T cell lymphoma (CTCL)[28]. Our results show that HDAC2/6 is among the functional DEGs in H5N1-infected A549 cells, supporting a possible role for vorinostat in reversal of H5N1-induced ALI.

Kelch-like ECH-associated protein 1 (KEAP1) has been implicated in LPS-induced ALI via regulation of the Nrf2/Keap1 pathway[29]. Our data revealed KEAP1, the molecular target of DMF, a drug approved for multiple sclerosis and psoriasis, as a functional DEG in H5N1-infected A549 cells. These results suggest a possible role for the Nrf2/Keap1 pathway in the observed action of DMF against H5N1-induced ALI. Digoxin is a cardiovascular medicine that increases the free calcium concentration by inhibiting the sodium/potassium-transporter ATPase subunit alpha-1 (ATP1A1)[30]. ATP1A1 has been associated with lung injury in mice[31], and its expression was significantly changed in cultured cells infected with H5N1 in this study. Our results indicate that H5N1-induced ALI in mice might be reduced by the ATP1A1 inhibitor digoxin. ALI in H5N1-infected mice was also attenuated by cladribine, a drug used to treat lymphoproliferative disorders, and the antimalarial agent pyrimethamine[32, 33]. The exact mechanisms of these actions need to be further studied.

In this study, we examined only 41 commercially available drugs from a total of 59 approved drugs identified when we overlapped the results from our high-throughput RNA sequencing of H5N1-infected A549 cells with the DrugBank database. The remaining 18 drugs need to be further studied, as do drug candidates currently under development in clinical trials.

In summary, we developed a highly effective, economic and safe method for drug repositioning and identified novel potential therapeutics for influenza virus A H5N1 infection. This approach may be generalized to discover candidates for drug repurposing to prevent and treat other diseases.

## Materials and methods

### Ethics statement

The animal experiments in this work were approved by the Ethics Committee of the Institute of Basic Medical Sciences, Chinese Academy of Medical Sciences (ACUC-A02-2015-003). All experimental protocols followed the Chinese National Guidelines.

### Viruses, cell culture and drugs

We performed *in vitro* and *in vivo* experiments using live influenza viruses A/Jilin/9/2004 (H5N1) and A/New Caledonia/20/1999 (H1N1) in biosafety level 3 facilities at the Institute of Military Veterinary Medicine, Academy of Military Sciences (Changchun, China). A549 human lung adenocarcinoma epithelial cells (American Type Culture Collection, Rockville, MD, USA) were cultured in Ham's F12 nutrient medium; Madin-Darby canine kidney (MDCK) cells were cultured in DMEM (HyClone, Logan, UT, USA). Streptomycin and penicillin (100 U ml^−1^) and fetal bovine serum (10%, Gibco, Grand Island, NY, USA) were added into culture medium. Test drugs were purchased from Selleck, Sigma-Aldrich or Med Chem Express.

### Cell viability assays

A549 cells were infected with live H1N1 influenza virus at a multiplicity of infection (MOI) of 4, live H5N1 influenza virus (MOI = 4) or an equal volume of vehicle (allantoic fluid; AF). The MTS [(3-(4,5-dimethylthiazol-2-yl)-5-(3-carboxymethoxyphenyl)-2-(4-sulfophenyl)-2H-tetrazolium), Promega, G3582, WI, USA] assay was used to determine the viability of A549 cells at a series of time points, 0, 15 min, 30 min, 1 h, 2 h, 3 h, 6 h, 9 h, 12 h, 18 h, 24 h, 36 h, and 48 h, after infection.

In some experiments with H5N1-infected A549 cells, the cells were exposed to drug candidates at 1 h before infection (to assess prophylactic efficacy) or at 3 h after infection (to assess therapeutic efficacy); cell viability was measured by the MTS assay at 48 h after infection.

### Quantitative real-time PCR

Total RNA was isolated using TRIzol Reagent (Invitrogen, CA, USA). Complementary DNA (cDNA) was synthesized from 1.5 μg of total RNA using a High Capacity cDNA Reverse Transcription Kit (Applied Biosystems). Quantitative real-time PCR was performed using a LightCycler 480 PCR System and the corresponding SYBR Green I Master kit (Roche, Basel, Switzerland). Influenza A virus matrix 2 (M2) gene expression levels were normalized against GAPDH (glyceraldehyde 3-phosphate dehydrogenase) in A549 cells and to β-actin in mouse lung tissue samples. The following primers were used: M2 forward, 5′- ATTGTGGATTCTTGATCGTC-3′; M2 reverse, 5′TGACAAAATGACCATCGTC-3′; human GAPDH forward, 5′-CGGAGTAACGGATTTGGTC-3′; human GAPDH reverse, 5′-TGGGTGGAATCATATTGGAACAT-3′; mice β-actin forward, 5′-CTCTCCCTCACGCCATCC-3′; mice β-actin reverse, 5′-CGCACGATTTCCCTCTCAG-3′).

### Animal experiments

Six-week-old wild-type C57BL/6 mice (Vital River, Beijing, China) were intratracheally instilled with live H5N1 virus (10^6^ TCID_50_) and given drugs or vehicle (control) 3 and 24 h before infection and at 24 h after infection. Detailed information about the drug dosage and product information is shown in S2 Table. Mice were sacrificed three days after infection. The lungs were removed from the thoracic cavity, collected in glass containers with approximately 50 mL of fixative, and treated with standard processes described previously[7]. Pathology images after hematoxylin and eosin staining were examined by three independent pathologists. For each mouse, 100 microscopic fields were analyzed to calculate lung injury scores and infiltrating cell numbers[34]. Pulmonary edema was assessed using the wet to dry weight ratio.

### Viral titration

Five C57BL/6 mice per group were administered drugs or vehicle at 3 and 24 h before and at 24 h and 48 h after infection (10^6^ TCID_50_ H5N1 virus). Detailed information about the drug dosage and product information is shown in S2 Table. Mouse lung tissues were collected and homogenized in PBS at 3 days after infection. MDCK cells were inoculated with 10-fold dilutions of homogenates in a 96-well plate, and infected cells were maintained in culture for 72 hours. The Reed-Muench method was used to calculate virus titers.

### Mice survival and loss of body weight

C57BL/6 mice (n = 6–10 per group) were infected with 10^6^ TCID_50_ H5N1 virus and intraperitoneally injected with vehicle, FAD (100 mg/kg), amitriptyline HCl (45 mg/kg), azacitidine (10 mg/kg) or calcitriol (0.1 mg/kg) four times: at 24 and 3 h before and at 24 and 48 h after infection. The rates of survival and loss of body weight were daily recorded until 15 days after infection.

### RNA-seq

Total RNA from human A549 cells and lung tissues of H5N1-infected mice exposed to drugs or control was isolated using TRIzol Reagent (Invitrogen, USA). A549 cell line samples were collected at 15 min, 30 min, 1 h, 2 h, 3 h, 6 h, 9 h, 12 h, 18 h, 24 h, 36 h, and 48 h after infection with H1N1 or H5N1. Lung tissues were collected at 2 d after H5N1 instillation. High-throughput, strand-specific RNA-seq (paired-end, 100 bp, 10 GB for each sample) was performed using the Illumina HiSeq2500 platform (Berry Genomics, Beijing).

### RNA-seq data analysis

Strand-specific paired-end RNA-seq was performed. FastQC (version 0.11.2) was used to control the quality of RNA-seq reads. We used Bowtie2 (version 2.1.0) and Tophat2 (version 2.0.11) to map RNA-seq reads to the human genome (version hg19, http://hgdownload.cse.ucsc.edu/downloads.html) and mouse genome (version mmc10), respectively. Cufflinks (version 2.2.1), Cuffmerge (version 2.2.1), and Cuffdiff (version 2.2.1) software were used to assemble transcription units, calculate gene expression levels (Fragments Per Kilobase of transcript per Million fragments mapped, FPKM value), and identify genes differentially expressed genes between samples.

### Gene functional enrichment analysis

Metacore (Clarivate Analytics, USA) software was employed to analyze functional pathways and processes for differentially expressed genes. Cytoscape (version 3.6.1) software with the Enrichment Map application (version 3.1.0) was used for process visualization. R software (version 3.5.1, www.r-project.org/) with the package pheatmap (version 1.0.10, cran.r-project.org/web/packages/pheatmap/) was utilized for heatmap production. We considered two-tailed P values < 0.05 and Benjamini-adjusted P values < 0.05 to be statistically significant.

### Drug candidate screening

The DrugBank database (version 4.3, www.drugbank.ca/) was used to screen drug candidates by comparing drug target genes and differentially expressed genes (DEGs). Detailed information about the drug manufacturers and product specifications are shown in S2 Table. The flowchart of drug candidate screening was as follows:

1. The “XML” file, which contained the information for all the approved small molecule drugs recorded in the DrugBank database, was downloaded, and target information was extracted using python software (version 2.6.6, https://www.python.org/).
2. Pathway enrichment analysis of DEGs from the comparison between virus-infected and uninfected cell models was performed; the detailed process is described in the “Gene functional enrichment analysis” section.
3. DEGs involved in significantly enriched pathways were extracted as functional DEGs, and redundant DEGs were removed to obtain unique functional DEGs.
4. Overlap between the unique functional DEGs filtered from our RNA-seq data and the drug target genes extracted from the DrugBank database was assessed, whereby drug candidates were considered those with targets that were also unique functional DEGs.

### Statistical analysis

A one-sample Kolmogorov-Smirnov test was used to confirm the normal distribution of the samples. We used a multiple comparison test with the Holm-Sidak method or ANOVA tests to compare groups. Pearson linear correlation analysis was applied to analyze the relationship between the number of DEGs and cell viability or viral replication in virus-infected A549 cells. The log-rank test was used to analyze the Kaplan-Meier survival curves. R software (version 3.5.1, www.r-project.org/) was used to control the false discovery rate (FDR) for Benjamini & Hochberg multiple testing correction. A two-tailed P value < 0.05 was considered statistically significant.

## Supporting information

S1 Fig Flowchart of screening for drug repurposing. Metacore database was used for functional enrichment; DrugBank database was used for searching target genes of approved drugs.
(TIF)

S2 Fig Candidate drugs against H5N1 in the A549 cell line. Viabilities of A549 cells based on the MTS assay at 48 h after H5N1 virus infection. Cells were treated with drugs or vehicle (control) either at 1 h before infection or at 3 h after infection. Data are presented as the mean ± SEM. All experiments were repeated at least twice. *P<0.05, **P<0.01, ***P<0.001 (two-tailed multiple comparison t-test with Holm-Sidak method, n = 3 biological replicates). Detailed information about *in vitro* drug treatment is shown in S2 Table.
(TIF)

S3 Fig Efficacy of drugs against H5N1 infection in mice. **(A)** Flowchart of screening for drugs in mice infected with H5N1 (10^6^ TCID_50_) via intratracheal instillation. **(B)** Wet to dry weight ratios (mean ± SEM) of mouse lungs at 3 d after infection and with gavage administration of drugs (n = 4–6 mice per group). All experiments were performed at least twice. **(C)** Body weight changes (mean ± SEM) of H5N1-infected mice treated with FAD (no. 2), amitriptyline HCl (no. 13), azacitidine (no. 14), and calcitriol (no. 18) or vehicle by intraperitoneal injection.
(TIF)

S4 Fig Functional pathways influenced by effective drugs in H5N1-infected mice. **(A)** Flowchart for RNA sequencing of lung tissues from drug-treated mice at 2 d after infection. **(B)** Heatmaps of RNA sequencing data showing the numbers of objects related to traditional drug indications or a repurposed indication of lung-related disease in functional enrichment pathways of mouse lung tissue. Pathways with a two-tailed P value < 0.05 and multiple-testing Benjamini & Hochberg correction < 0.05 were considered significant. Abbreviations: LN, lung neoplasm; LI, lung disease (interstitial); LO, lung disease (obstructive); PF: pulmonary fibrosis; T, traditional indication-related disease. Detailed information about pathways and diseases related objects in the pathways is shown in S7–S13 Tables.
(TIF)

S1 Table Top 3 pathway enrichment clusters in H1N1/H5N1-infected A549 cells. (XLSX)

S2 Table Drug information. (XLSX)

S3 Table Traditional/Lung disease-related objects in the Top 5 pathways with amitriptyline HCl (No.13) administration. (XLSX)

S4 Table Traditional/Lung disease-related objects in the Top 5 pathways with FAD (No. 2) administration. (XLSX)

S5 Table Traditional/Lung disease-related objects in the Top 5 pathways with azacitidine (No. 14) administration. (XLSX)

S6 Table Traditional/Lung disease-related objects in the Top 5 pathways with calcitriol (No. 18) administration. (XLSX)

S7 Table Traditional/Lung disease-related objects in the Top 5 pathways with digoxin (No. 6) administration. (XLSX)

S8 Table Traditional/Lung disease-related objects in the Top 5 pathways with bosutinib (No. 17) administration. (XLSX)

S9 Table Traditional/Lung disease-related objects in the Top 5 pathways with cladribine (No. 20) administration. (XLSX)

S10 Table Traditional/Lung disease-related objects in the Top 5 pathways with DMF (No. 23) administration. (XLSX)

S11 Table Traditional/Lung disease-related objects in the Top 5 pathways with pyrimethamine (No. 29) administration. (XLSX)

S12 Table Traditional/Lung disease-related objects in the Top 5 pathways with ruxolitinib (No. 30) administration. (XLSX)

S13 Table Traditional/Lung disease-related objects in the Top 5 pathways with vorinostat (No. 33) administration. (XLSX)

## References

[1] Van Norman GA. Overcoming the Declining Trends in Innovation and Investment in Cardiovascular Therapeutics: Beyond EROOM's Law. JACC Basic to translational science. 2017;2(5):613–25. 10.1016/j.jacbts.2017.09.002 30062175 PMC6058942

[2] Nosengo N. Can you teach old drugs new tricks? Nature. 2016;534(7607):314–6. 10.1038/534314a 27306171

[3] Bauer TT, Ewig S, Rodloff AC, Muller EE. Acute respiratory distress syndrome and pneumonia: a comprehensive review of clinical data. Clinical infectious diseases: an official publication of the Infectious Diseases Society of America. 2006;43(6):748–56. 10.1086/506430 PMC7107989 16912951

[4] Peiris JS, Cheung CY, Leung CY, Nicholls JM. Innate immune responses to influenza A H5N1: friend or foe? Trends in immunology. 2009;30(12):574–84. 10.1016/j.it.2009.09.004 19864182 PMC5068224

[5] Perwitasari O, Yan X, O'Donnell J, Johnson S, Tripp RA. Repurposing Kinase Inhibitors as Antiviral Agents to Control Influenza A Virus Replication. Assay Drug Dev Technol. 2015;13(10):638–49. 10.1089/adt.2015.0003.drrr 26192013 PMC4692129

[6] Yan Y, Zou Z, Sun Y, Li X, Xu KF, Wei Y, et al Anti-malaria drug chloroquine is highly effective in treating avian influenza A H5N1 virus infection in an animal model. Cell research. 2013;23(2):300–2. 10.1038/cr.2012.165 23208422 PMC3567830

[7] Zou Z, Yan Y, Shu Y, Gao R, Sun Y, Li X, et al Angiotensin-converting enzyme 2 protects from lethal avian influenza A H5N1 infections. Nature communications. 2014;5:3594 10.1038/ncomms4594 24800825 PMC7091848

[8] Li C, Yang P, Sun Y, Li T, Wang C, Wang Z, et al IL-17 response mediates acute lung injury induced by the 2009 pandemic influenza A (H1N1) virus. Cell research. 2012;22(3):528–38. 10.1038/cr.2011.165 22025253 PMC3292301

[9] Wang W, Yang P, Zhong Y, Zhao Z, Xing L, Zhao Y, et al Monoclonal antibody against CXCL-10/IP-10 ameliorates influenza A (H1N1) virus induced acute lung injury. Cell research. 2013;23(4):577–80. 10.1038/cr.2013.25 23419516 PMC3616436

[10] de Chassey B, Meyniel-Schicklin L, Aublin-Gex A, Andre P, Lotteau V. Genetic screens for the control of influenza virus replication: from meta-analysis to drug discovery. Molecular bioSystems. 2012;8(4):1297–303. 10.1039/c2mb05416g 22307679

[11] Lesch M, Luckner M, Meyer M, Weege F, Gravenstein I, Raftery M, et al RNAi-based small molecule repositioning reveals clinically approved urea-based kinase inhibitors as broadly active antivirals. PLoS pathogens. 2019;15(3):e1007601 10.1371/journal.ppat.1007601 30883607 PMC6422253

[12] Pizzorno A, Terrier O, Nicolas de Lamballerie C, Julien T, Padey B, Traversier A, et al Repurposing of Drugs as Novel Influenza Inhibitors From Clinical Gene Expression Infection Signatures. Frontiers in immunology. 2019;10:60 10.3389/fimmu.2019.00060 30761132 PMC6361841

[13] Ekins S, Nikolsky Y, Bugrim A, Kirillov E, Nikolskaya T. Pathway mapping tools for analysis of high content data. Methods Mol Biol. 2007;356:319–50. 10.1385/1-59745-217-3:319 16988414

[14] Law V, Knox C, Djoumbou Y, Jewison T, Guo AC, Liu Y, et al DrugBank 4.0: shedding new light on drug metabolism. Nucleic Acids Res. 2014;42(Database issue):D1091–7. 10.1093/nar/gkt1068 24203711 PMC3965102

[15] Flierl MA, Rittirsch D, Nadeau BA, Chen AJ, Sarma JV, Zetoune FS, et al Phagocyte-derived catecholamines enhance acute inflammatory injury. Nature. 2007;449(7163):721–5. 10.1038/nature06185 17914358

[16] Al-Harbi NO, Imam F, Nadeem A, Al-Harbi MM, Korashy HM, Sayed-Ahmed MM, et al Riboflavin attenuates lipopolysaccharide-induced lung injury in rats. Toxicology mechanisms and methods. 2015;25(5):417–23. 10.3109/15376516.2015.1045662 26360969

[17] Li Q, Wall SB, Ren C, Velten M, Hill CL, Locy ML, et al Thioredoxin Reductase Inhibition Attenuates Neonatal Hyperoxic Lung Injury and Enhances Nuclear Factor E2-Related Factor 2 Activation. American journal of respiratory cell and molecular biology. 2016;55(3):419–28. 10.1165/rcmb.2015-0228OC 27089175 PMC5023024

[18] Britt RD Jr., Velten M, Locy ML, Rogers LK, Tipple TE. The thioredoxin reductase-1 inhibitor aurothioglucose attenuates lung injury and improves survival in a murine model of acute respiratory distress syndrome. Antioxidants & redox signaling. 2014;20(17):2681–91. 24295151 10.1089/ars.2013.5332 PMC4026403

[19] Samanta S, Zhou Z, Rajasingh S, Panda A, Sampath V, Rajasingh J. DNMT and HDAC inhibitors together abrogate endotoxemia mediated macrophage death by STAT3-JMJD3 signaling. The international journal of biochemistry & cell biology. 2018;102:117–27. 30010012 10.1016/j.biocel.2018.07.002 PMC6309960

[20] Thangavel J, Samanta S, Rajasingh S, Barani B, Xuan YT, Dawn B, et al Epigenetic modifiers reduce inflammation and modulate macrophage phenotype during endotoxemia-induced acute lung injury. Journal of cell science. 2015;128(16):3094–105. 10.1242/jcs.170258 26116574 PMC4541045

[21] Shi YY, Liu TJ, Fu JH, Xu W, Wu LL, Hou AN, et al Vitamin D/VDR signaling attenuates lipopolysaccharideinduced acute lung injury by maintaining the integrity of the pulmonary epithelial barrier. Molecular medicine reports. 2016;13(2):1186–94. 10.3892/mmr.2015.4685 26675943 PMC4732862

[22] Xu J, Yang J, Chen J, Luo Q, Zhang Q, Zhang H. Vitamin D alleviates lipopolysaccharideinduced acute lung injury via regulation of the reninangiotensin system. Molecular medicine reports. 2017;16(5):7432–8. 10.3892/mmr.2017.7546 28944831 PMC5865875

[23] Fang W, Cai SX, Wang CL, Sun XX, Li K, Yan XW, et al Modulation of mitogenactivated protein kinase attenuates sepsisinduced acute lung injury in acute respiratory distress syndrome rats. Molecular medicine reports. 2017;16(6):9652–8. 10.3892/mmr.2017.7811 29039541

[24] Qian F, Deng J, Wang G, Ye RD, Christman JW. Pivotal Role of Mitogen-Activated Protein Kinase-Activated Protein Kinase 2 in Inflammatory Pulmonary Diseases. Current protein & peptide science. 2016;17(4):332–42. 26119506 10.2174/1389203716666150629121324 PMC4878395

[25] Song Z, Zhao X, Gao Y, Liu M, Hou M, Jin H, et al Recombinant human brain natriuretic peptide ameliorates trauma-induced acute lung injury via inhibiting JAK/STAT signaling pathway in rats. The journal of trauma and acute care surgery. 2015;78(5):980–7. 10.1097/TA.0000000000000602 25909419

[26] Wu J, Yan X, Jin G. Ulinastatin protects rats from sepsis-induced acute lung injury by suppressing the JAK-STAT3 pathway. Journal of cellular biochemistry. 2018. 10.1002/jcb.27550 30242880

[27] Ni YF, Wang J, Yan XL, Tian F, Zhao JB, Wang YJ, et al Histone deacetylase inhibitor, butyrate, attenuates lipopolysaccharide-induced acute lung injury in mice. Respiratory research. 2010;11:33 10.1186/1465-9921-11-33 20302656 PMC2848144

[28] Mann BS, Johnson JR, Cohen MH, Justice R, Pazdur R. FDA approval summary: vorinostat for treatment of advanced primary cutaneous T-cell lymphoma. The oncologist. 2007;12(10):1247–52. 10.1634/theoncologist.12-10-1247 17962618

[29] Sun CY, Xu LQ, Zhang ZB, Chen CH, Huang YZ, Su ZQ, et al Protective effects of pogostone against LPS-induced acute lung injury in mice via regulation of Keap1-Nrf2/NF-kappaB signaling pathways. International immunopharmacology. 2016;32:55–61. 10.1016/j.intimp.2016.01.007 26800098

[30] The effect of digoxin on mortality and morbidity in patients with heart failure. The New England journal of medicine. 1997;336(8):525–33. 10.1056/NEJM199702203360801 9036306

[31] Leikauf GD, Concel VJ, Bein K, Liu P, Berndt A, Martin TM, et al Functional genomic assessment of phosgene-induced acute lung injury in mice. American journal of respiratory cell and molecular biology. 2013;49(3):368–83. 10.1165/rcmb.2012-0337OC 23590305 PMC3824050

[32] Tetreault SA, Robbins BA, Saven A. Treatment of hairy cell leukemia-variant with cladribine. Leukemia & lymphoma. 1999;35(3–4):347–54. 10706459 10.3109/10428199909145739

[33] Doberstyn EB, Phintuyothin P, Noeypatimanondh S, Teerakiartkamjorn C. Single-dose therapy of falciparum malaria with mefloquine or pyrimethamine-sulfadoxine. Bulletin of the World Health Organization. 1979;57(2):275–9. 373903 PMC2395771

[34] Matute-Bello G, Downey G, Moore BB, Groshong SD, Matthay MA, Slutsky AS, et al An official American Thoracic Society workshop report: features and measurements of experimental acute lung injury in animals. American journal of respiratory cell and molecular biology. 2011;44(5):725–38. 10.1165/rcmb.2009-0210ST 21531958 PMC7328339

