# Supplementary figures and images for: Identification of amitriptyline HCl, flavin adenine dinucleotide, azacitidine and calcitriol as repurposing drugs for influenza A H5N1 virus-induced lung injury
Source: PLoS Pathog. 2020 Mar 16;16(3):e1008341. doi: 10.1371/journal.ppat.1008341 (PMC7075543; doi:10.1371/journal.ppat.1008341)

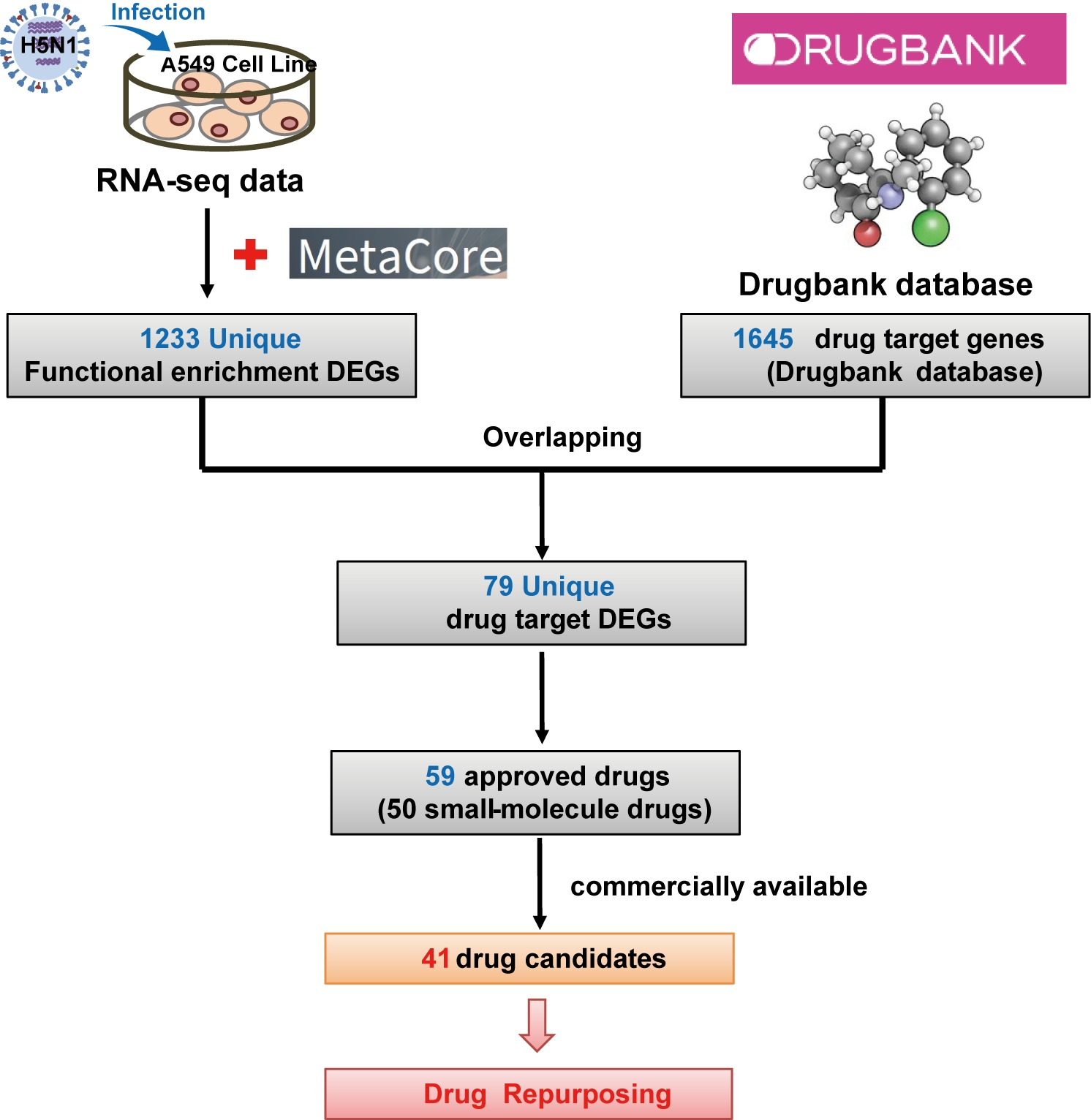

Supplement: S1 Fig — Metacore database was used for functional enrichment; DrugBank database was used for searching target genes of approved drugs. (TIF) [file ppat.1008341.s001.tif]

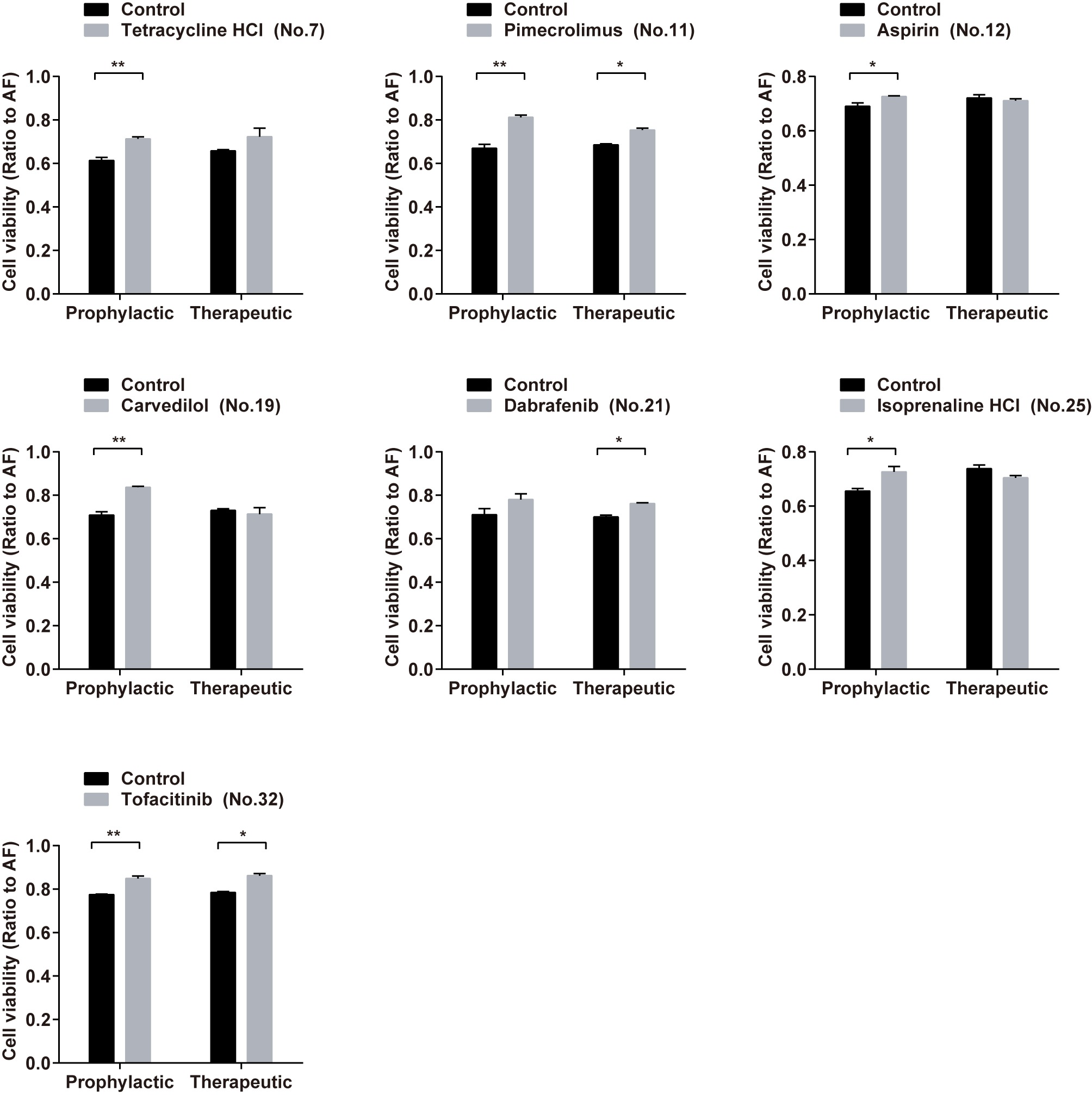

Supplement: S2 Fig — Viabilities of A549 cells based on the MTS assay at 48 h after H5N1 virus infection. Cells were treated with drugs or vehicle (control) either at 1 h before infection or at 3 h after infection. Data are presented as the mean ± SEM. All experiments were repeated at least twice. *P<0.05, **P<0.01, ***P<0.001 (two-tailed multiple comparison t-test with Holm-Sidak method, n = 3 biological replicates). Detailed information about in vitro drug treatment is shown in S2 Table. (TIF) [file ppat.1008341.s002.tif]

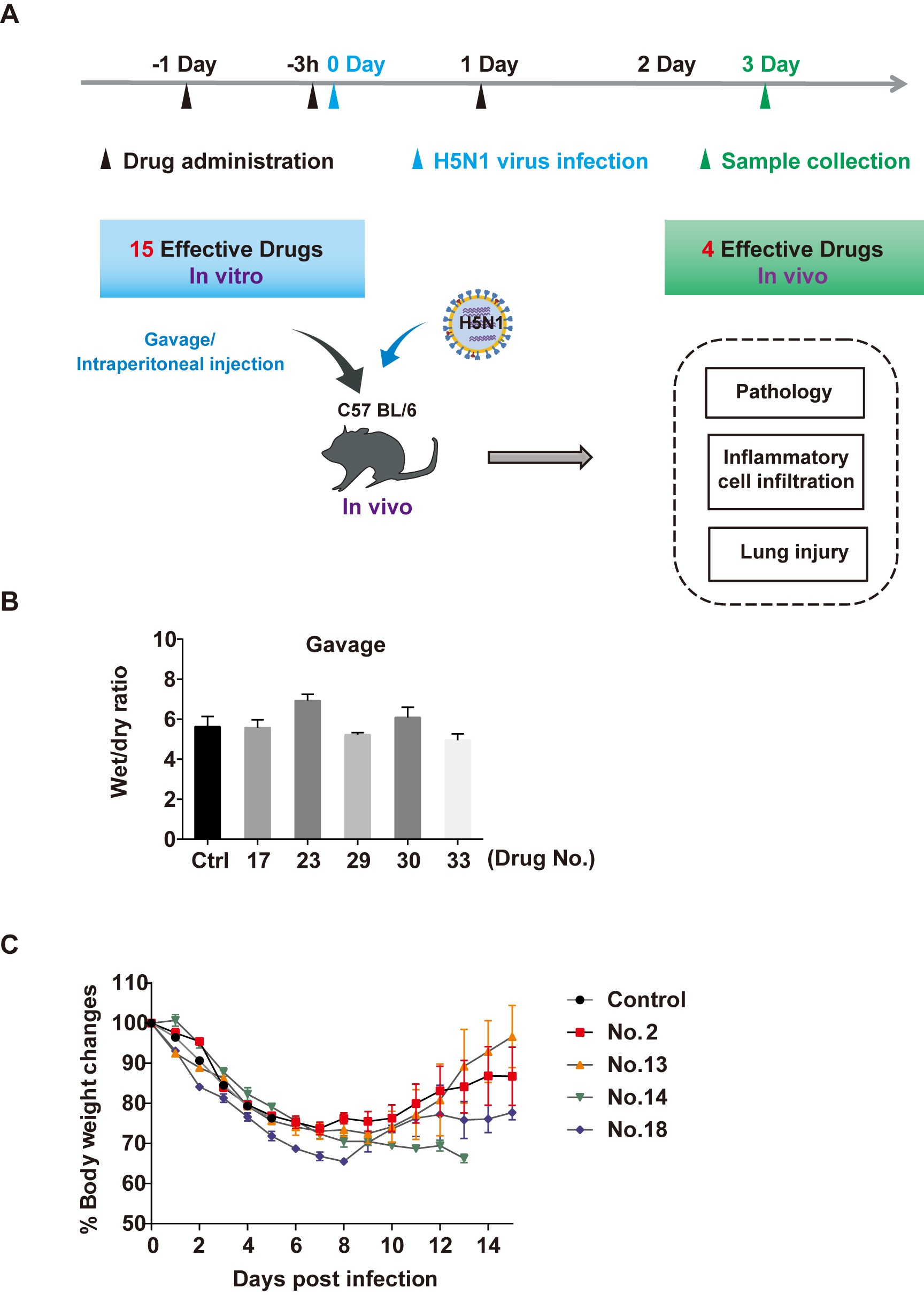

Supplement: S3 Fig — (A) Flowchart of screening for drugs in mice infected with H5N1 (106 TCID50) via intratracheal instillation. (B) Wet to dry weight ratios (mean ± SEM) of mouse lungs at 3 d after infection and with gavage administration of drugs (n = 4–6 mice per group). All experiments were performed at least twice. (C) Body weight changes (mean ± SEM) of H5N1-infected mice treated with FAD (no. 2), amitriptyline HCl (no. 13), azacitidine (no. 14), and calcitriol (no. 18) or vehicle by intraperitoneal injection. (TIF) [file ppat.1008341.s003.tif]

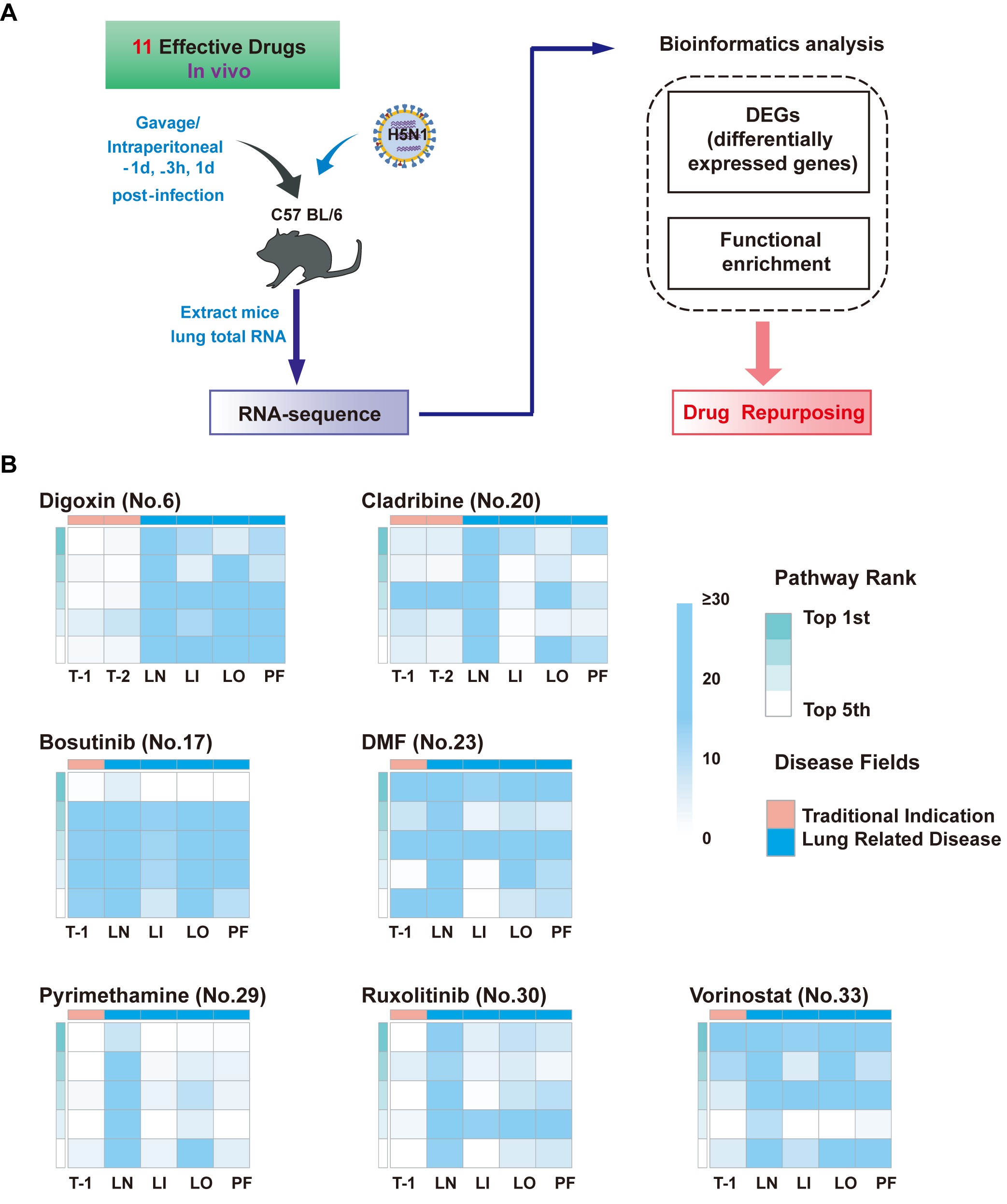

Supplement: S4 Fig — (A) Flowchart for RNA sequencing of lung tissues from drug-treated mice at 2 d after infection. (B) Heatmaps of RNA sequencing data showing the numbers of objects related to traditional drug indications or a repurposed indication of lung-related disease in functional enrichment pathways of mouse lung tissue. Pathways with a two-tailed P value < 0.05 and multiple-testing Benjamini & Hochberg correction < 0.05 were considered significant. Abbreviations: LN, lung neoplasm; LI, lung disease (interstitial); LO, lung disease (obstructive); PF: pulmonary fibrosis; T, traditional indication-related disease. Detailed information about pathways and diseases related objects in the pathways is shown in S7–S13 Tables. (TIF) [file ppat.1008341.s004.tif]
